# Floral Induction in the Short-Day Plant Chrysanthemum Under Blue and Red Extended Long-Days

**DOI:** 10.3389/fpls.2020.610041

**Published:** 2021-01-25

**Authors:** Malleshaiah SharathKumar, Ep Heuvelink, Leo F. M. Marcelis, Wim van Ieperen

**Affiliations:** Horticulture and Product Physiology, Wageningen University and Research, Wageningen, Netherlands

**Keywords:** blue extended long-day, chrysanthemum, photoperiodic flowering, morphology, supplemental lighting, vertical farm

## Abstract

Shorter photoperiod and lower daily light integral (DLI) limit the winter greenhouse production. Extending the photoperiod by supplemental light increases biomass production but inhibits flowering in short-day plants such as *Chrysanthemum morifolium*. Previously, we reported that flowering in growth-chamber grown chrysanthemum with red (R) and blue (B) LED-light could also be induced in long photoperiods by applying only blue light during the last 4h of 15h long-days. This study investigates the possibility to induce flowering by extending short-days in greenhouses with 4h of blue light. Furthermore, flower induction after 4h of red light extension was tested after short-days RB-LED light in a growth-chamber and after natural solar light in a greenhouse. Plants were grown at 11h of sole source RB light (60:40) in a growth-chamber or solar light in the greenhouse (short-days). Additionally, plants were grown under long-days, which either consisted of short-days as described above extended with 4h of B or R light to long-days or of 15h continuous RB light or natural solar light. Flower initiation and normal capitulum development occurred in the blue-extended long-days in the growth-chamber after 11h of sole source RB, similarly as in short-days. However, when the blue extension was applied after 11h of full-spectrum solar light in a greenhouse, no flower initiation occurred. With red-extended long-days after 11h RB (growth-chamber) flower initiation occurred, but capitulum development was hindered. No flower initiation occurred in red-extended long-days in the greenhouse. These results indicate that multiple components of the daylight spectrum influence different phases in photoperiodic flowering in chrysanthemum in a time-dependent manner. This research shows that smart use of LED-light can open avenues for a more efficient year-round cultivation of chrysanthemum by circumventing the short-day requirement for flowering when applied in emerging vertical farm or plant factories that operate without natural solar light. In current year-round greenhouses’ production, however, extension of the natural solar light during the first 11 h of the photoperiod with either red or blue sole LED light, did inhibit flowering.

## Introduction

Flowering time is governed by various internal and external factors including developmental competence, circadian rhythms, temperature, and photoperiod (Srikanth and Schmid, 2011; Cho et al., 2017). Many plant species monitor seasonal changes in the light environment (photoperiod, light intensity, direction, and spectral composition) to optimize their growth and development (Thomas, 2006). Photoperiod influences floral induction and flowering rate in many flowering plant species. Based on photoperiod requirement plants are classified into short-day (SD) and long-day (LD) plants (Garner and Allard, 1920). However, short- and long-night plants would be more accurate as it is the length of the dark period that is decisive for flower induction (Borthwick et al., 1952). The perception of photoperiod takes place in leaves *via* photoreceptors that are well described in model plant species (Song et al., 2015). Additionally, differences in the light spectrum are perceived by a distinct set of photoreceptors; red/far-red [phytochromes (PHY)], blue/UV-A [cryptochromes (CRY)], [phototropins (PHOT), ZTL/FKF_1_/LKP_2_], and UV-B light (UVR8). Most of the flowering plants possess several of these photoreceptors and together these photoreceptors influence and regulate flowering, through a complex network of regulatory genes (Andrés and Coupland, 2012; Viczián et al., 2020). Photoperiodic flowering is controlled, in part, by light signals that entrain the circadian clock, which is an essential component of the mechanism for day-length sensing by plants and is involved in the regulation of flowering as explained by the “external coincidence” model for flowering (Johansson and Staiger, 2015). The control of photoperiodic flowering operates by upregulation of florigen – *FLOWERING LOCUS T* (*FT*) and downregulation of anti-florigenic *FT* (*AFT*) / *TERMINAL FLOWER 1* (*TFL1*) under inductive photoperiod and this mechanism is conserved in both LD and SD plants (Higuchi, 2018).

In addition to photoperiod, light spectrum plays a regulatory role in flowering in both short-day and long-day plants (Cerdán and Chory, 2003; Song et al., 2015). In many long-day species, blue and far-red light accelerates flower induction (Song et al., 2015; Zhang et al., 2020), and the presence of far-red light during the daily photoperiod or given at end-of-day accelerates flowering (Lane et al., 1965; Vince, 1965; Runkle and Heins, 2003). Long-day plants grown under a far-red deficient environment delayed floral initiation and development in crops such as lisianthus, snapdragon (van Haeringen et al., 1998), tussock bellflower (*Campanula carpatica*), tickseed (*Coreopsis grandifora*; Runkle and Heins, 2001), and petunia (*Petunia hybrida*; Kim et al., 2002). In the short-day plant’s such as poinsettia (Zhang and Runkle, 2019), garden strawberry (Fragaria × ananassa), and chrysanthemum illumination with end-of-day far-red delayed flowering (Hisamatsu et al., 2008). Red light is typically effective in inhibiting the flowering of short-day plants. Various photoperiod studies demonstrated that a red light night-break could inhibit flowering of cocklebur (*Xanthium strumarium*), soybean (*Glycine max*), and chrysanthemum, and a subsequent far-red exposure could reverse the flowering inhibition (Borthwick et al., 1952; Downs, 1956; Cathey and Borthwick, 1957; Thomas and Vince-Prue, 1996). Similar flowering inhibition was also observed in dahlia (*Dahlia hortensis*), and marigold (*Tagetes erecta*) under 4h night-break by red (Craig and Runkle, 2013). Furthermore, the combination of far-red with red light, delivered as night-break, were effective both for inhibiting flowering in short-day plant (marigold) and for promoting flowering of long-day plants (petunia and snapdragon; Craig and Runkle, 2013). Besides red and far-red, blue light is known for flower promoting effects (Guo et al., 1998; Song et al., 2012). Blue light delivered as night-break or daylength-extension promoted flowering of long-day plants compared to the short-days (Goto et al., 1991; Yamada et al., 2011; Lopez et al., 2020), whereas in the short-day plants perilla (*Perilla ocymoides*) and rice (Hamamoto et al., 2003; Ishikawa et al., 2009), its delayed flowering. Therefore, flowering responses vary depending on the quality of light, photoperiodic lighting (daylength-extension or night-break), and on species.

Chrysanthemum is a commercially important species that occupies a large share of the global market of cut-flower production (Higuchi, 2018). To meet the global demand for marketable flowers throughout the year, the flowering time of this obligate short-day plant is highly regulated by supplemental lighting by daylength-extension or by night-breaks to prevent premature flowering (Higuchi et al., 2013; Park and Jeong, 2020). For many years, most of the light spectrum studies on chrysanthemum flowering regulation are confined to the effect of the light spectrum during night-breaks (Cathey and Borthwick, 1957; Borthwick and Cathey, 1962; Kadman-Zahavi and Ephrat, 1971, 1973; Horridge and Cockshull, 1989; Higuchi et al., 2012; Liao et al., 2014; Park and Jeong, 2020). Supplemental lighting is also used for photosynthesis and growth enhancement during short days, while it is particularly needed to avoid substantial flowering delays when daily light integrals are low (Langton, 1992). An earlier study in a growth chamber experiment in our lab demonstrated the possibility of inducing flowering under long-days (15h) by extending red-blue short-days with 4h of photosynthetic active blue light (Jeong et al., 2014). Such a treatment would be highly interesting if it could be used in the commercial greenhouse industry, where solar light instead of red-blue light is present during the short-day period. This is not certain as it has been shown that the composition of the light spectrum during the short-day period may alter the night-break flowering responses to light spectrum in chrysanthemum (Higuchi et al., 2012). Therefore, in the present study, we investigated whether it is possible to induce flowering by extending short-days to long-days with 4h of blue LED light after short-days of natural full-spectrum daylight in greenhouses. Additionally, flower induction after 4h of red daylight extension was tested after short-days of RB (growth-chamber) or natural full-spectrum daylight (greenhouse).

## Materials and Methods

### Plant Material and Growth Conditions

Peat block-rooted cuttings of *Chrysanthemum morifolium* cv. “Radost” (Deliflor Chrysanten B.V, Netherlands) were transplanted in 8cm × 8cm × 10cm plastic pots containing a peat-based horticultural substrate (Lentse Potgrond, Horticoop), which contains 810gm^−3^ N-P-K in the ratio of 15-10-20 and had a pH = 5.7 and EC = 0.8dSm^−1^. The transplanted cuttings were placed in a greenhouse and grown with a 15h long-day photoperiod (solar light) for 7days. The day/night temperature was 23/18 ± 4°C, the relative humidity 60–72%, and the CO_2_ concentration was ambient. Water was supplied every other day *via* overhead irrigation. After 7days, the plants were moved to the experimental greenhouse or the growth-chamber with the final spectral light treatments and grown for 6weeks. Each spectral light treatment had 125 plants including 34 border plants. Realized day/night temperatures were 22/18°C ± 2°C (greenhouse) and 20/18°C ± 0.2°C (growth-chamber). Relative humidity was 60–72% (greenhouse) and 65 ± 2% (growth-chamber). CO_2_ concentration was ambient. To achieve uniform climate conditions, six small electric fans per light treatment were installed in each plot in the growth-chamber. Plants were irrigated *via* overhead irrigation every other day, with a nutrient solution (Hoagland, pH = 5.9 ± 0.2, EC = 1.2dS m^−1^).

### Lighting Treatments

**Figure 1:**
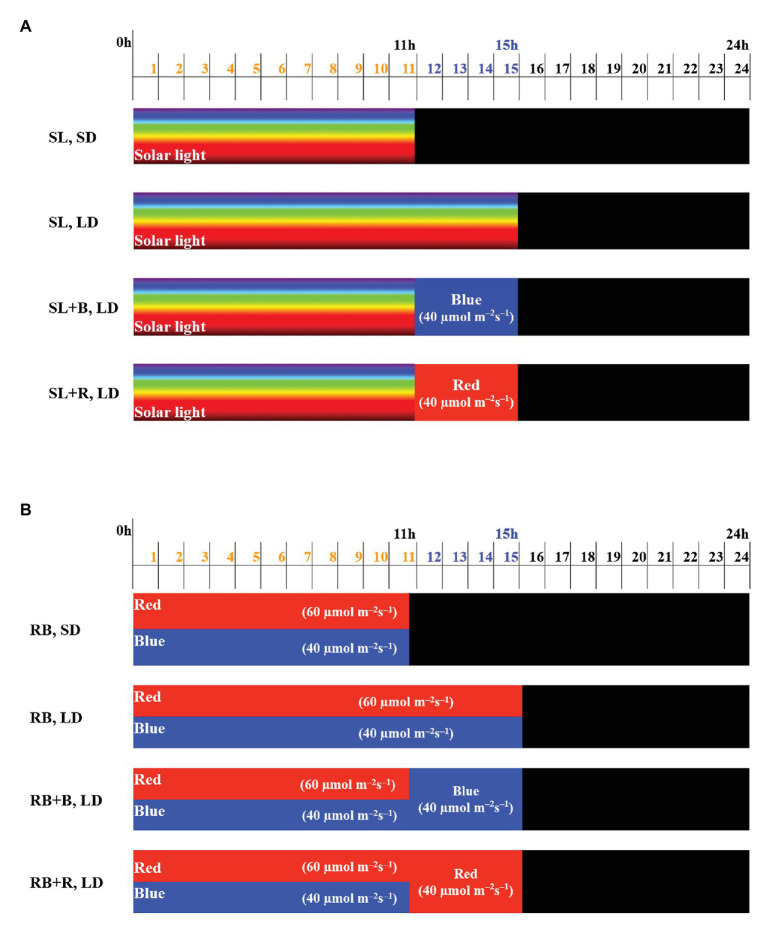
Schematic representation of light treatments applied in **(A)** greenhouse or **(B)** climate chamber. Multicolor or red or blue colors indicate day light period; black color indicates dark period. The numbers in parenthesis indicate the light intensities supplied by red and blue LEDs (μmolm^−2^ s^−1^). SL light intensity in the greenhouse varied between and within days according to season and weather.

**Figure 2:**
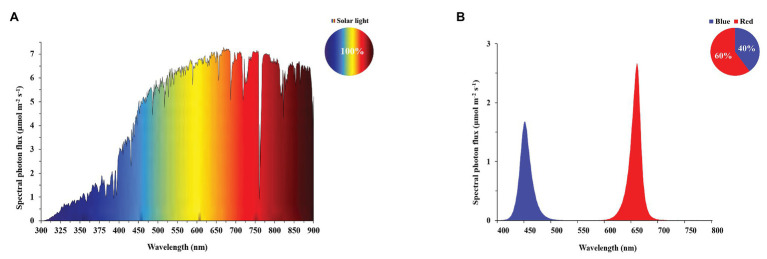
Spectral photon distribution of **(A)** solar light, measured at a representative day in the greenhouse at noon, and **(B)** measured in a mixed RB treatment in the growth chamber. The separate spectral photon distributions in R and B reflect the wavelength distribution of the supplied narrow-band R and B light during daylength extensions in the greenhouse as well as in the growth chamber.

**Greenhouse Experiment:** Four light treatments were applied, in which in each treatment the photoperiod started with 11h of natural full-spectrum solar light (SL; Figure 1A): (1) solar light, SD – 11h of natural full-spectrum solar light; (2) solar light, LD – 15h of natural full-spectrum solar light; (3) solar light + B, LD – 11h of natural full-spectrum solar light, extended by 4h of blue light; and (4) solar light + R, LD – 11h of natural full-spectrum solar light, extended by 4h of red light. Obviously, the light intensity during the first 11h of each light treatment (during 15h in the solar LD treatment) varied with solar irradiance outside the greenhouse. Incidental light measurements at plant level in experimental plots indicated light integrals that were of the same order of magnitude as in the growth chamber experiment (described below). Detailed global solar radiation data over the full experimental period (measured outside the greenhouse) are provided in the Supplementary Material. The greenhouse compartment was divided into 16 plots of 1.0 × 1.3m^2^. Light treatments were repeated four times randomized over these 16 plots. To avoid light interference between light treatments, we used double-layered white plastic screens. The experiment was executed during summer, and to achieve a precise photoperiod of short-day (11h) and long-day (15h), solar light was blocked by black-out screens. To achieve the red or blue light day extensions, plants were illuminated by red or blue LEDs (Signify GreenPower LEDs research modules) with a peak wavelength of 450 (blue) and 660nm (red; Figure 2). LEDs were positioned ∼1m above the plants. The photosynthetic photon flux density (PPFD) during blue or red light photoperiod extension was 40μmolm^−2^ s^−1^. The LED light intensity was kept constant at plant height by adjusting twice per week to correct for an increase in plant height. Light spectra (Figure 2) and PPFD of LED light were measured using a spectroradiometer (Specbos 1211, Jeti Technische Instrumente GmbH, Jena, Germany). Solar light intensity in the greenhouse varied between and within days according to season and weather.

**Growth-Chamber Experiment:** Four light treatments were applied, in which in each treatment the photoperiod started with 11h of red (R) and blue (B) light mixture at a 60:40 ratio (Figure 1B): (1) RB, SD – 11h of mixed red and blue light; (2) RB, LD – 15h of mixed red-blue light; (3) RB+B, LD – 11h of mixed red and blue light, extended by 4h of blue light; and (4) RB+R, LD – 11h of mixed red and blue light, extended by 4h of red light. Light treatments were repeated four times simultaneously in three different climate rooms (four treatments in each of two climate rooms, four treatments in two replicates in the same climate room in parallel). To avoid light interference among light treatment, we used double-layered white plastic screens between plots. Custom-built illumination systems containing red and blue LEDs (Signify GreenPower LEDs research modules) were used with a peak wavelength of either 450 (blue) or 660nm (red; Figure 2). Illumination systems were suspended ∼1m above the plants. All plants received a PPFD of 100 ± 5μmolm^−2^ s^−1^ during the first 11h of each light treatment and 40μmolm^−2^ s^−1^ during red or blue light day extensions. The PPFD during the RB, LD treatment was 100μmolm^−2^ s^−1^. The PPFD was kept constant at plant height by adjusting twice per week to correct for plant growth.

### Flowering Observations

**Figure 3:**
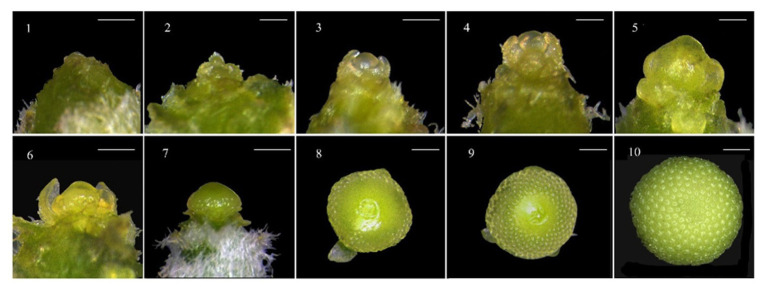
Developmental stages of floral transition in *Chrysanthemum morifolium* cv. Radost. Stage-1 (vegetative phase), − the flat shoot apical meristem (SAM). Stage-2, − between the leaf primordia, a barely perceptible bulge of SAM happens. Stage-3, − the bulge is larger and the leaf primordia begin to deviate. Stage-4, − the first bracts are visible under the leaf primordia. Stage-5, − the bracts cover the dome. Stage-6 (floral initiation phase) – first floral primordia visible. Stage-7 (floral development phase) – one to four rings of floral primordia visible. Stage-8, − multiple rings of floral primordia visible (<half of area of flower head). Stage-9, − multiple rings of floral primordia visible (>half of area of flower head); Stage-10, − entire bud covered with floral primordia (visible bud emerges). Images were taken from a stereoscope with 80.0x magnification. When the diameter of apex was more than 2mm (image 8–10), a lower magnification (from 30 to 70 x) was used to fit whole apex in the field of view. White lines at the left top side of each image indicate the length of 1mm.

The developmental stages of chrysanthemum shoot apical meristem (SAM) up to the visible flower bud stage were microscopically examined and described (Figure 3). To detect the number of days for floral initiation, every other day stereoscopic SAM dissections were conducted on two randomly selected plants per light treatment from day 8 until day 28 after the start of light treatments. Three centimeter long shoot apices were excised and immediately dissected to reach the SAMs by carefully peeling off leaves and removing leaf primordia with a surgical knife under a stereoscope. Images of developing stages of SAMs were acquired on a Zeiss Stereo Discovery-V12 microscope equipped with a Plan S 1.0 lens and an Axiocam MRc5 camera controlled by Axio Vision 4.8.1.0 software. Dissected SAMs with distinctive developmental stages of floral transition were imaged to assess the number of days for floral initiation. Floral initiation was confirmed when the SAM attained floral developmental stage 6 (first floral primordia initiation stage; Figure 3). Based on the linear regression between flower developmental stage and time, it was deduced when stage 6 was reached. The obtained value was considered as the number of days taken for floral initiation. Derived values for each of the light treatments were subjected to one-way ANOVA. Daily recording of the number of days for visible flower bud appearance started 8days after the start of the light treatments on 10 plants per plot. The number of buds per plant and flowering (%) were recorded until day 42. Additionally, 10 plants were used to follow capitulum development and anthesis until day 55.

### Growth and Morphology Observations

Growth and morphology were measured on day 42 after the onset of light treatments. Ten plants per plot were used to record stem length (cm), number of internodes and leaves and the leaf area (LI-COR 3100 area meter). Dry weights of leaves, stem, and flower buds were measured after oven-drying at 105°C for 24h and used to calculate the total shoot dry weight.

### Statistical Design and Analysis

In both experiments, four-light treatments were arranged in a randomized design over 16 plots. Hence, four replicate plots were used per light treatment. Out of 125 plants per plot, 34 were border plants, for SAM dissections, two plants per observation day starting from day 8 to day 28 (22 plants per plot), and on day 42, 10 plants per plot were used for growth and morphology observations, and on day 55, 10 plants per plot were used for observing flower capitulum. All statistical analyses were performed using Genstat (18th edition; VSN International Ltd., Herts, United Kingdom). One-way ANOVA, according to a complete randomized design, was applied to test for light treatment effect (*p* = 0.05). Mean separation was done by Fisher’s Protected LSD test (*p* = 0.05).

## Results

### Short-Day Light Spectrum Influenced Flowering Under Red and Blue Extended Long-Days

**Figure 4:**
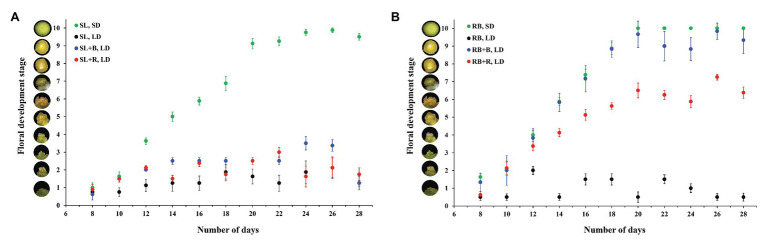
Effect of different light treatments on floral initiation of *Chrysanthemum morifolium* cv. Radost. Floral initiation and development of shoot apical meristem in greenhouse **(A)** and climate room **(B)** scored as per the floral developmental stages of Chrysanthemum (see the Figure 3), stage 6 is considered as floral initiation attainment [data are represented as mean ± SE (*n* = 8)].

**Figure 5:**
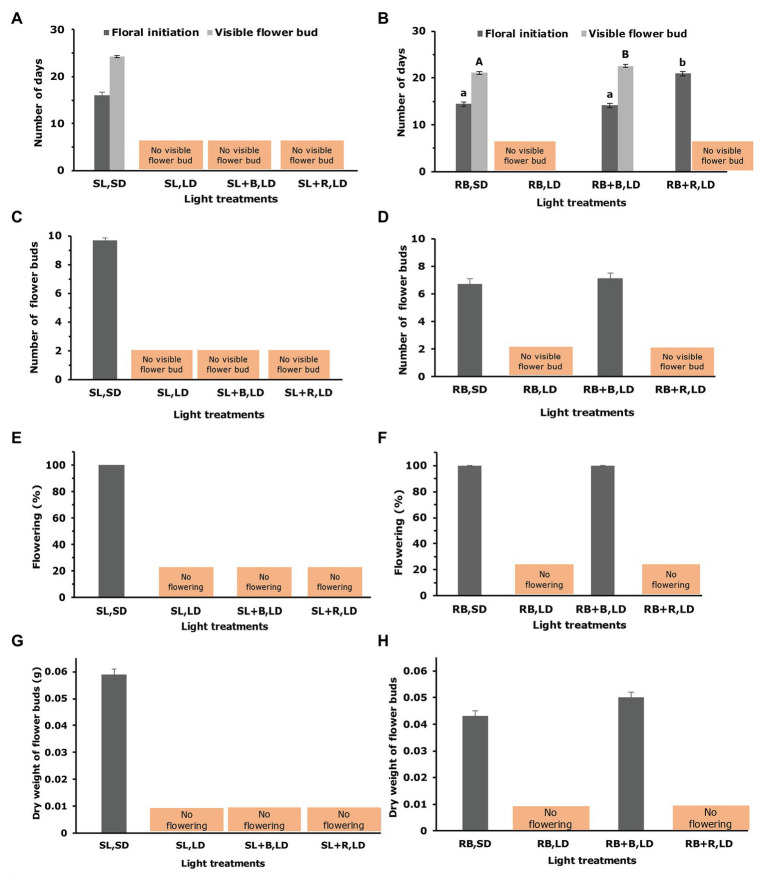
Effect of different light treatments on flowering response of *Chrysanthemum morifolium* cv. Radost. Number of days until floral initiation and visible flower buds **(A,B)**; number of flower buds per plant **(C,D)**; percentage of flowering plants **(E,F)**; dry weight of flower buds **(G,H)** on the 42th day after start of light treatments. Panel **(A,C,E,G)** under solar light in greenhouse experiment and panel **(B,D,F,H)** under sole source red-blue LEDs lighting in growth chamber experiment [data are represented as mean ± SE (*n* = 10)]. Different letters indicate that means differed significantly (Fisher’s Protected LSD test, *p* = 0.05). No letters indicate that means not differed significantly. Light treatment label details: see Figure 1.

**Figure 6:**
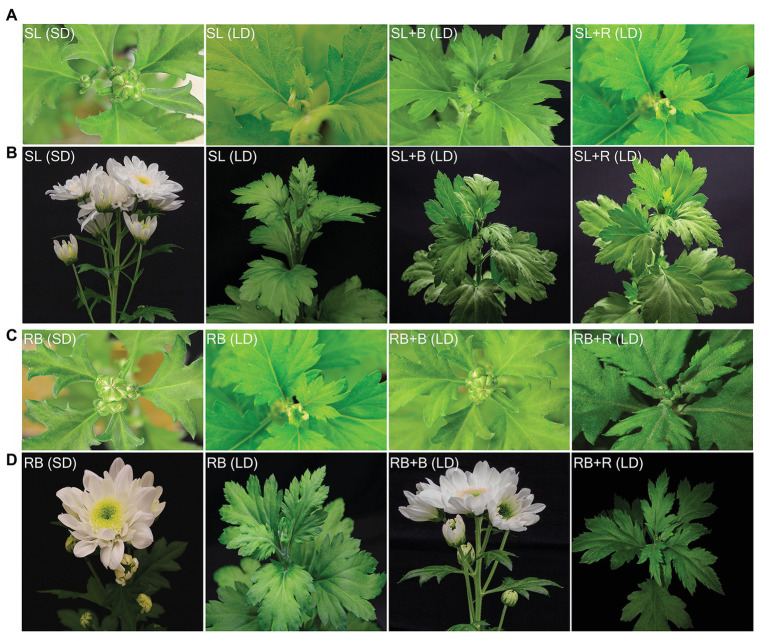
Flower buds **(A,C)** and flower capitulum **(B,D)** of *Chrysanthemum morifolium* cv. Radost on 25 **(A,C)** and 55 **(B,D)** days after start of eight different light treatments at different photoperiods and spectral composition. Panel **(A,B)** under solar light (SL) in greenhouse experiment and panel **(C,D)** sole source red-blue LEDs lighting (RB) in growth chamber. The label in each image denotes the specific light treatment, with comparable daylength and spectral composition (in case of daylength extension) in the same column.

Under constant sole source red and blue lighting and natural solar light, chrysanthemum flowered in short-days and not in long-days (Figures 4, 5). Extending the short-days of solar light in a greenhouse with either blue or red light to long-days did not result in flowering (Figures 4A, 5A), while the same daylight extensions (with either red or blue) after a short-day with sole source red and blue light resulted in floral initiation (Figure 4). However, full capitulum development and anthesis occurred only when these short-days were extended with blue light (Figures 6C,D). All plants, which were grown under red-blue short-day (RB, SD) and red-blue short days extended to long days with blue light (RB + B, LD) reached the floral initiation within 14days from the start of the light treatment, while the plants that were grown under red-blue short-days extended with red light (RB + R, LD) reached the final floral initiation stage 5–6days later (Figure 5B). Plants grown under 11h red-blue extended with 4h blue succeeded in attaining visible bud stage in 22–23days, which was only 1–2days later than in red-blue short-day (Figures 5B, 6C) and produced the same number of flower buds as plants grown under red-blue short-day (Figure 5D). Flower buds of RB, SD and RB + B, LD plants weighed almost equal (Figure 5H). All plants, which were grown under short-day (red-blue and solar light) and blue extended long-day (RB + B, LD) recorded 100% flowering (Figures 5E,F).

### Plant Morphology and Growth Under Red and Blue Extended Long-Days

**Figure 7:**
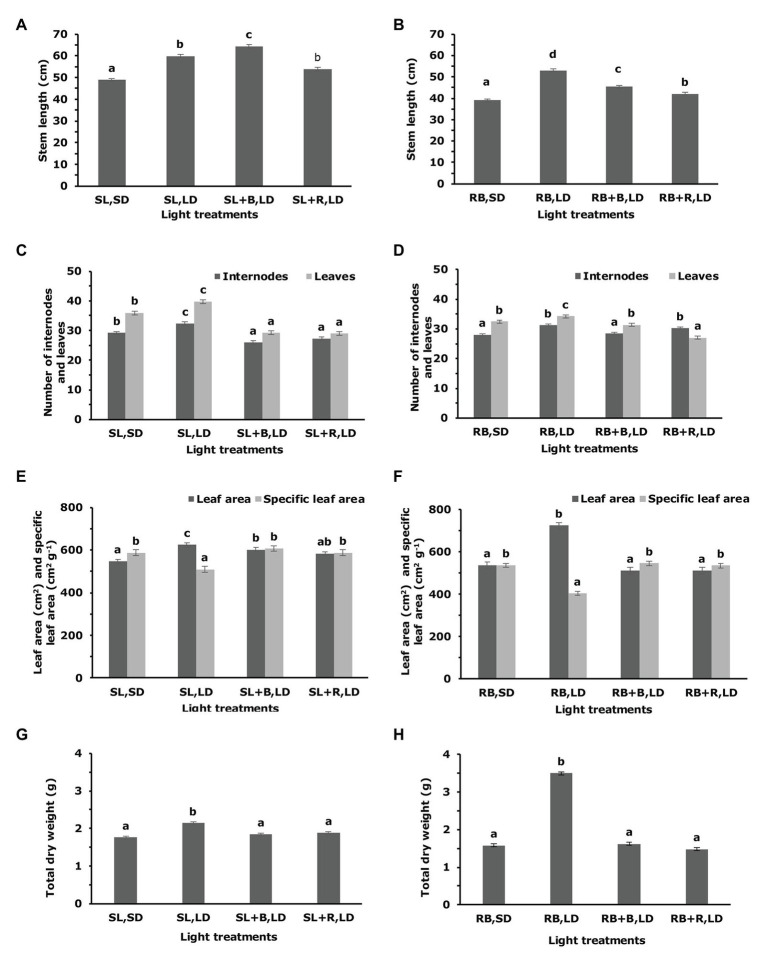
Effect of spectrally different daylength extensions on plant growth of *Chrysanthemum morifolium* cv. Radost in a greenhouse and growth chamber. Stem length **(A,B)**; number of internodes and leaves **(C,D)**; leaf area and specific leaf area **(E,F)**; total dry weight **(G,H)**; on the 42th day after start of light treatments. Panel **(A,C,E,G)** under solar light in greenhouse experiment and panel **(B,D,F,H)** under sole source re-blue LEDs lighting in growth chamber experiment [data are represented as mean ± SE (*n* = 10)]. Different letters indicate that means differed significantly (Fisher’s Protected LSD test, *p* = 0.05). Light treatment label details: see Figure 1.

In the greenhouse experiment, stem length was higher in plants that were grown under solar light extended with blue light (SL + B, LD) than under solar light short-day (SL, SD) and solar light extended with red light (SL + R, LD; Figure 7A). The number of internodes and leaves was higher in solar light long-day treatments compared to solar light short-day and solar light extended with 4h of either blue or red light treatments (Figure 7C). Plants grown under 15h long-day solar light photoperiod had a larger leaf area compared to 11h short-day photoperiod (Figure 7E). Under 15h solar light, the specific leaf area was lower than under solar light short-day and solar light extended with 4h of either blue or red light treatments (Figure 7E). The total dry weight did not exhibit a significant difference between solar light short-day and photoperiods extended by red and blue, but plants grown under solar light LD had higher total dry weight (Figure 7G).

Some of the morphological responses were slightly different between the greenhouse and the climate chamber experiments. Increasing the photoperiod from 11h sole source red-blue light to 15h always resulted in an increased stem length, as was also observed in the greenhouse experiment, but the contrasts among the long-day treatments differed from the results obtained in the greenhouse (Figure 7B). The number of internodes and leaves in the blue and red extended long-day treatments (RB + R, LD and RB + B, LD) was not lower than in the short-day (RB, SD) treatment (Figure 7D), as was the case in the greenhouse experiment under solar light. In the climate chamber, only the extension of the day with red-blue light resulted in a larger leaf area (RB, LD; Figure 7F), whereas in the greenhouse experiment leaf area increased in all long-day treatments compared to the short-day treatment. Specific leaf area showed similar responses as in the greenhouse experiment and was lower in the short-day treatment than the long-day treatments. Similar to the greenhouse experiment, the only long-day treatment with a higher total dry weight at harvest was the normal long-day (RB, LD), but the relative increase was larger than in the greenhouse (Figure 7H).

## Discussion

Growing short-day chrysanthemum in 11h of red-blue extended with 4h of monochromatic blue (100% artificial light) resulted in flowering despite the 15h long photoperiod (Figures 5B, 6C,D). This confirms the earlier study by our lab (Jeong et al., 2014). However, plants grown in a greenhouse under 11h of solar light extended with 4h of monochromatic blue or red light failed to flower (Figures 5A, 6A,B). This could have been due to several aspects that differed in the light climates between the growth chamber and greenhouse, among which differences in light intensity and differences in spectral composition of the light received by the plants during light periods before the day-length extension (the first 11h of each light period).

Last decade, important steps were made in unraveling molecular mechanisms underlying photoperiodic flowering in Chrysanthemum: altered light signals influence the signal transduction pathway of important flower regulatory genes in *Chrysanthemum morifolium* (floral inhibiting antiflorigen *CmAFT* and floral stimulating florigen *CmFTL3*) of which the expression levels are clock regulated, daylength dependent, and control photoperiodic flowering (Oda et al., 2012; Higuchi et al., 2013). Much of this progress is the result of loss-of-function studies in the diploid *C. seticuspe*, which is much easier to transform, than the hexaploid *C. morifolium* that is commonly used in commercial production of chrysanthemum. However, even in *C. seticuspe*, it is still largely uncertain, how *CsFTL3* and *CsAFT* are regulated by light to define the critical night length for flowering (Oda et al., 2020).

### Potential Effects of Light Intensity Differences Between Greenhouse and Growth Chamber

An adequate carbon supply is vital for developmental transitions, such as flowering in plants, and can be sensed through sugar signaling (Wingler, 2018). In Arabidopsis, for instance, trehalose-6-phosphate (T6P) is involved in sugar status sensing and also required for the expression of FT and flowering (Wahl et al., 2013). Differences in light intensity between the growth chamber and greenhouse experiments influencing carbon availability to the plants might therefore be important. Other than in the growth chamber experiment, the light intensity in the greenhouse varied with natural solar light over and between the days (see Supplementary Material). The daily light integrals of photosynthetic active radiation (DLI) outside the greenhouse were much higher than the DLIs in the growth chamber, but due to the low transmissivity of the research greenhouse, and the use of light screens to avoid stray light between plots, light intensity in the greenhouse plots was strongly reduced. Because the overall transmissivity variated with the changing angle of incidence of solar radiation over the day, it is impossible to estimate DLIs at plant level from the outside radiation measurements. Incidental measurements in the greenhouse plots, conducted around noon, yielded light intensities that were slightly higher than those in the growth chamber plots (results not shown). However, based on the almost similar dry weight of the plants after 42days of growth in the SD-treatments in the greenhouse and in the growth chamber (Figures 7G,H), it is reasonable to assume that the total light integrals in the growth chamber and greenhouse experiment were not very different. It is well-known that low light integral delays flower initiation and retards flower development in chrysanthemum. Langton (1992) showed that the number of inductive SD’s required for flowering exponentially increases below 4.6–6.9mol PAR m^−2^ d^−1^ (depending on cultivar). The light integral in the growth chamber SD-treatment was approximately 4mol PAR m^−2^ d^−1^. Therefore, the very small difference in days until floral initiation between plants in the SD-treatments in greenhouse and growth chamber (Figures 5A,B) also indicate a not more than small difference in light integral. How low light integrals influence the timing of flowering in chrysanthemum still needs to be elucidated. It has been indicated by RNA-sequencing that sugar sensing through T6P might be involved in flowering of the summer-flowering chrysanthemum variety “Yuuka,” which flowers under SD and LD, though only under LDs (Ren et al., 2016). Later experiments with sucrose application on leaves supported this restriction to LDs, while in a strict SD-flowering variety, no effect of sucrose application on leaves on flowering was observed (Sun et al., 2017). Taking this all together, it is not very likely that there were substantial differences in light integral between the growth chamber and greenhouse experiments in the present study. Light integrals were low, but not that low that they strongly influenced the time to flowering. Additionally, the strong differences in response to the B-extended long days, with flowering in the growth chamber and no flowering in the greenhouse, occurred at substantially higher light integrals than in the SD-treatments, which both flowered.

### Flowering of Chrysanthemum in Long Photoperiods With Diurnal Spectra Variations

Previously it has been shown in chrysanthemum, that the light spectrum during a short photoperiod can strongly influence and even reverse the effectiveness of night-break of a certain color: blue or far-red night-breaks were effective in inhibiting flowering when plants were grown under a short photoperiod with monochromatic blue light, but not when they were applied after a short photoperiod with white light (Higuchi et al., 2012). Blue light increases the fraction of deactivated phytochrome similar to far-red (Sager et al., 1988). In the same study (Higuchi et al., 2012), the effect of far-red during night-break was exposure-time dependent and night-breaks with blue or far-red became ineffective in the inhibition of flowering when short day light spectrum was a mixture of red and blue light. Therefore, these authors suggested a role of at least two phytochromes (PHYA and PHYB) in the regulation of flowering in Chrysanthemum, but at the same time could not exclude a role for cryptochromes.

Interestingly, we observed that 11h of red-blue daylight extended with 4h of red triggered floral initiation, but that further capitulum development was arrested (Figures 4B, 5B), while a daylength extension with red in the greenhouse showed no stimulation of floral initiation at all (SL + R, LD). This shows, similar to comparing blue extended long days in climate room and greenhouse, that the light spectrum during the first 11h of the day period influences the effect of the light spectrum after the first 11h on flower initiation.

A large difference between solar light in the greenhouse and red-blue LEDs in the growth chamber during the first 11h of the photoperiod is the lack of green and far-red wavelengths in the LED-lighting. It may therefore be well possible that this lack of green and/or far-red is responsible for the different flowering responses in the growth chamber experiments compared to the greenhouse. Green light can influence the photoperiodic flowering of long-day and short-day plants (as reviewed by Zhang and Folta, 2012). The inhibitory effect of green light on flowering was shown by delivering green as a night-break in many short-day plants such as *Cosmos bipinnatus, Perilla ocymoides*, *Abelmoschus esculentus* and *Abelmoschus moschatus* ssp. *tuberosus* (Hamamoto et al., 2003; Hamamoto and Yamazaki, 2009), and chrysanthemum (Sumitomo et al., 2012). Likewise, chrysanthemum grown under a 12h white fluorescent light photoperiod extended with 4h of green (518nm) failed to flower (Jeong et al., 2012). Green light responses might be mediated by blue sensing cryptochromes (Zhang and Folta, 2012; Smith et al., 2017). In *Arabidopsis*, plants grown under simultaneous blue-green failed to flower, because the presence of green nullified the strong blue-induced flowering by reducing the *FT* levels. Green light reversed the blue-induced floral induction by CRY2 degradation and suppressing *FT* expression in *Arabidopsis* (Banerjee et al., 2007). In chrysanthemum, a 30min night-break with green (530nm) light delayed flowering by 17days compared to short-day by suppressing the expression of *CmFTL3* (Sumitomo et al., 2012). As of yet, it is not clear whether the presence of green light during the daylight spectrum interferes with the possible promotive effects of 4h of blue light day extension in chrysanthemum.

Another possible reason for non-flowering under short-days with solar light extended with either blue or red light could be a high fraction of far-red, during the photoperiod. Similar flowering inhibition due to higher far-red (735nm) light at the end of the day was reported in short-day plants, *Phabitis nil* (Fredericq, 1964) and rice (Ikeda, 1985). Far-red at the end of the day may be responsible for non-flowering due to altered phytochrome state. The inhibitory action of far-red light may be associated with the lowered level of phytochrome P_fr_ that is essential to start the dark reactions responsible in triggering the floral induction. In other terms, a high P_fr_ is needed for floral stimuli/florigen during inductive darkness (Higuchi et al., 2012). This inhibitory effect of far-red light was reported in other short-day plants such as duckweed (*Lemna paucicostata*) and *Xanthium pennsylvanicum* (Salisbury, 1965; Ohtani and Ishiguri, 1979). From our study, short-days of natural full-spectrum daylight followed by 4h blue or red day extension was obviously not enough to increase the amount of P_fr_ to stop floral inhibition. It can thus be suggested that the relative amounts of green or far-red during the daily photoperiod can possibly influence flowering genes to regulate photoperiodic flowering under solar light extended with 4h of blue or red day extension. Thus, chrysanthemum appears to be particularly sensitive to the spectral composition of 11h of daylight to flower under blue or red extended long-days. The present study suggests that, besides daylength, the spectral composition of the short-day photoperiod also influences the flowering responses.

### Growth and Morphology of Chrysanthemum in Long Photoperiods With Diurnal Spectra Variations

Extending the day with blue light promoted stem length due to internode elongation (Figures 7A,B), in agreement with Jeong et al. (2014). Similar effects of narrow-band blue light on stem elongation have been reported in other species such as petunia, salvia, and marigold (Heo et al., 2002; Fukuda et al., 2016). Narrow-band blue light is seen as a strong signal in enhancing shoot elongation, through modulation of gibberellin content (Fukuda et al., 2016). Stem elongation is strongly correlated with both internode appearance rate and internode elongation (Carvalho et al., 2002). Plants that were grown under short photoperiods (solar and red-blue) were shorter than their long-photoperiod counterparts in both growth environments (greenhouse and growth chamber). The length difference was caused by a lower number of internodes due to floral initiation, whereas average internode length was not affected (results not shown). The lower stem length in the RB + B, LD treatment compared to the RB long-day treatment can also be explained by a lower number of internodes due to flowering (Figures 7B,D) because the shift of the vegetative shoot apical meristem into a floral meristem stops the initiation of new leaves and new internodes on the main stem. Day-length extensions with R and B increased stem length compared to SDs in both the greenhouse and growth chamber experiments due to effects on internode elongation alone (Figures 7A,B), as in all day-extension treatments the number of internodes per stem remained similar (after 11h RB) or even slightly decreased (after 11h of SL).

Longer photoperiods increase the daily available light for the plant, which enhances total dry weight as observed under 15h of solar light and red-blue long-day photoperiod (Figures 7G,H). These results are consistent with Kurilčik et al. (2008), who reported a continuous increase in dry weight and leaf number in chrysanthemum with increased photoperiod duration from 8 to 24h. In contrast, to the observations made by Jeong et al. (2014) present results do not show a significant positive effect on dry weight in the blue extended long-day treatments, most likely due to the lower intensity of blue during day extension in present experiment. Similarly, stem length (an important quality attribute of chrysanthemum cut flower) differed between red-blue short-days and blue extended long-days (Jeong et al., 2014). Higher growth rate is often achieved by an increase in the net assimilation rate (Adams and Langton, 2005). Therefore, plants grown under red-blue long-day photoperiod showed higher total dry weight and leaf area compared to red-blue short-day and red-blue extended with either 4h of blue or red (Figures 7G,H). This is most likely because plants under red-blue extension received a higher PPFD of 100μmolm^−2^ s^−1^ compared to 40μmolm^−2^ s^−1^ under 4h of blue during daylight extension. Additionally, mixed red-blue light is known to increase total dry weight and leaf area in plants by increasing the net assimilation rate, compared to that by monochromatic blue or red light (Kim et al., 2004).

## Conclusion

Sole source red-blue short-day extended with 4h of sole blue resulted in complete flowering, while extension with 4h of sole red resulted in floral initiation but no further flower development took place. In contrast, plants in solar light short-day extended with 4h of blue or red light failed to flower. Our results show that, besides photoperiod, the spectral composition of the short-day part of the photoperiod influences the effect of the light spectrum thereafter on flowering. This limits the application of blue daylength extension in commercial greenhouse production of chrysanthemum. However, the smart use of LED-light opens up new avenues for a more efficient year round production of short-day plants in vertical farms or plant factories that operate without solar light.

Taken together, multiple components of the daylight spectrum may influence the mechanism of photoperiodic flowering in chrysanthemum in a time-dependent manner. Furthermore, more fundamental knowledge is needed about diurnal effects of light quality on the cascade of processes (from floral evocation to anthesis) to fully take advantage of the possibilities of LEDs in plant production systems. For this, the involvement of photoreceptors in the molecular framework of flowering regulatory genes such as florigen (*CmFTL3*) and antiflorigen (*CmAFT*) needs to be elucidated.

This study shows that not only day-length *per se* but also the spectral composition of the first 11h of a long photoperiod influences the flowering responses in chrysanthemum.

## Data Availability Statement

The raw data supporting the conclusions of this article will be made available by the authors, without undue reservation.

## Author Contributions

WI, EH, MS, and LM conceptualized the research idea and designed the experiments. MS performed the experiments and wrote the first version of the manuscript. MS, EH, and WI performed data analysis and all authors discussed the results and the interpretation of the data. MS, EH, and WI revised the manuscript according to the helpful comments of the reviewers. All authors contributed to the article and approved the submitted version.

### Conflict of Interest

The authors declare that the research was conducted in the absence of any commercial or financial relationships that could be construed as a potential conflict of interest.
